# Roux-en-Y Gastric Bypass Increases Intravenous Ethanol Self-Administration in Dietary Obese Rats

**DOI:** 10.1371/journal.pone.0083741

**Published:** 2013-12-31

**Authors:** James E. Polston, Carolyn E. Pritchett, Jonathan M. Tomasko, Ann M. Rogers, Lorenzo Leggio, Panayotis K. Thanos, Nora D. Volkow, Andras Hajnal

**Affiliations:** 1 Department of Neural and Behavioral Sciences, The Pennsylvania State University College of Medicine, Hershey, Pennsylvania, United States of America; 2 Department of Surgery, The Pennsylvania State University College of Medicine, Hershey, Pennsylvania, United States of America; 3 Section on Clinical Psychoneuroendocrinology and Neuropsychopharmacology, Laboratory of Clinical and Translational Studies, National Institute on Alcohol Abuse and Alcoholism (NIAAA), NIH, Bethesda, Maryland, United States of America; 4 Intramural Research Program, National Institute on Drug Abuse (NIDA), NIH, Baltimore, Maryland, United States of America; 5 Department of Behavioral and Social Sciences, Brown University, Providence, Rhode Island, United States of America; 6 Department of Psychology, Stony Brook University, Stony Brook, New York, United States of America; 7 Laboratory of Neuroimaging, NIAAA Intramural Program, NIH, Bethesda, Maryland, United States of America; INRA, France

## Abstract

Roux-en-Y gastric bypass surgery (RYGB) is an effective treatment for severe obesity. Clinical studies however have reported susceptibility to increased alcohol use after RYGB, and preclinical studies have shown increased alcohol intake in obese rats after RYGB. This could reflect a direct enhancement of alcohol’s rewarding effects in the brain or an indirect effect due to increased alcohol absorption after RGYB. To rule out the contribution that changes in alcohol absorption have on its rewarding effects, here we assessed the effects of RYGB on intravenously (IV) administered ethanol (1%). For this purpose, high fat (60% kcal from fat) diet-induced obese male Sprague Dawley rats were tested ∼2 months after RYGB or sham surgery (SHAM) using both fixed and progressive ratio schedules of reinforcement to evaluate if RGYB modified the reinforcing effects of IV ethanol. Compared to SHAM, RYGB rats made significantly more active spout responses to earn IV ethanol during the fixed ratio schedule, and achieved higher breakpoints during the progressive ratio schedule. Although additional studies are needed, our results provide preliminary evidence that RYGB increases the rewarding effects of alcohol independent of its effects on alcohol absorption.

## Introduction

The growing epidemic of obesity and associated health consequences represents a major cause of preventable death. Roux-en-Y gastric bypass (RYGB) is an effective treatment for severe obesity, with factors other than restriction and malabsorption likely contributing to the effect [1], [2]. RYGB patients voluntarily restrict consumption of sugar- and fat-rich palatable foods [3], [4], [5], [6]. RYGB reduces appetite despite reduced caloric intake and weight loss. Recent studies demonstrate attenuated preference for sugars and fats following RYGB, possibly by reducing food reward [6], [7], [8]. Conversely, reports of increased risk for ethanol (EtOH) consumption following RYGB have raised concerns that RYGB may increase the vulnerability for alcohol use disorder [9], [10]. Notably, RYGB patients have higher and longer-lasting blood EtOH concentrations, and a shorter period of onset than non-surgical controls when consuming similar amounts of EtOH [11], [12], [13], [14]. Changes in EtOH’s absorption and pharmacokinetics may alter not only the bioavailability and stimulating properties of EtOH on the brain, but also influence the neuronal and hormonal signals upstream of the reward system. However, the extent of which RYGB contributes to the increase in alcohol reward independent of the changes in EtOH absorption and pharmacokinetics is unclear.

Our group recently showed that high fat diet-induced obese (DIO) rats that underwent RYGB rats consumed twice as much EtOH as sham-operated obese controls, and 50% more than normal-diet lean controls [15]. RYGB also increased the breakpoint for EtOH operant responding, indicating an increased willingness to work for EtOH reward following surgery [16]. A recent report from another laboratory, using a slightly different surgical RYGB technique and a different strain of outbred rats (i.e., Long Evans vs. Sprague Dawley), also showed increased EtOH preference in DIO rats after RYGB [17]. However, given that in these studies EtOH was administered orally, it was not possible to differentiate if the effects were due to a direct enhancement of EtOH’s rewarding effects, or to an indirect effect brought about by changes in EtOH’s absorption and consequent bioavailability and pharmacokinetics [18].

Here we used an intravenous (IV) operant model of EtOH self-administration to evaluate direct changes in alcohol reward without the potential confound of changes in EtOH’s absorption. We used fixed ratio (FR) and progressive ratio (PR) schedules of reinforcement to evaluate if RYGB modified the reinforcing effects of EtOH. We hypothesized that RYGB would increase EtOH’s rewarding effects (even when administered IV), thus increasing overall EtOH self-administration and animals’ willingness to seek and work for EtOH.

## Methods

### Ethics Statement

All experiments were carried out in strict accordance with the recommendations in the Guide for the Care and Use of Laboratory Animals of the National Institutes of Health and were approved by the Pennsylvania State University College of Medicine Institutional Animal Care and Use Committee.

### Subjects

Twelve adult (4 week old) male Sprague Dawley rats (Charles River, Wilmington, MA), with starting weights between 250–275 g were housed in individual temperature- and humidity-controlled cages and maintained on a 12∶12-hr light-dark cycle (lights on at 0700). Rats were water-restricted during the initial habituation period (2 days) to the self-administration continuous access-licking task, receiving water for 3 hrs after habituation sessions (daily; afternoon).

### Diet and Alcohol

All rats received a nutritionally complete high fat diet (#D12492; Research Diets, New Brunswick, NJ) consisting of 5.24 kcal/gram (60% kcal fat, 20% kcal carbohydrates and 20% kcal protein) for at least 26 weeks prior to and throughout the study. Alcohol (95%, Pharmco Products Inc., CT) was diluted in deionized water to 1% v/v, prepared daily.

### Roux-en-Y Gastric Bypass Surgery

After 26–28 weeks on a high fat diet, animals received either RYGB or SHAM. The techniques and perioperative care have been described previously [16]. Briefly, rats fasted overnight, with water ad lib prior to surgery. Anesthetized rats (isofluorane: 3% for induction, 1.5% for maintenance) were pretreated with antibiotic (Ceftriaxone: 100 mg/kg, im; Roche, Nutley, NJ). Sterilely, through a midline laparotomy, the stomach was divided in the RYGB procedure to create a smaller gastric pouch separated from the bypassed stomach using a linear-cutting stapler (ETS-Flex Ethicon Endo surgery, 45 mm). The jejunum was divided 15 cm from the ligament of Treitz. The distal segment was anastomosed end-to-side to form a pouch gastrojejunostomy. The proximal jejunum was anastomosed end-to-side 15 cm along the distal limb. Anastomoses were created with interrupted 5–0 polypropylene sutures. Abdominal wall and skin were closed using 3–0 silk and 5–0 nylon. Sham controls received gastric manipulation as if a stapler were to be inserted, and then replaced to its normal location, then a transverse enterotomy 15 cm from the ligament of Treitz, reclosed with 5–0 polypropylene sutures. Local anesthetic (0.5 ml of 0.25% bupivacaine, sc.) was used to minimize postoperative pain. Postoperative care included normal saline (50 ml/kg, sc.) immediately before and after surgery, and on postoperative day 1, and buprenorphine (0.5 mg/kg, im) given as needed for pain. After 24 hrs, animals received BOOST® (Nestle Nutrition, Minneapolis, MN) and ad lib water for 3 days. On postoperative day 3, the animals returned to their high fat diet.

### Self-Administration Catheters and Jugular Implantation

Approximately two months after RYGB or SHAM, rats were anesthetized with isoflurane and surgically implanted with catheters into the right external jugular vein, routed subcutaneously to the back and attached to a coupling assembly as previously described [19]. Syringe pumps were connected to a swivel system in the test chambers, enabling computer controlled IV infusion of EtOH (1%). Catheters were flushed daily with 0.2 ml of heparinized saline to maintain patency, and verified, as needed, using 0.2 ml of IV propofol (Diprivan 1%) intravenously. Following catheterization, subjects recovered for five days.

### Apparatus

Testing took place in one of six identical operant chambers (MED Associates, St. Albans, VT) in a separate testing environment. Ethanol delivery was triggered by a lickometer circuit, in which licks on an empty bottle (the active spout) triggered an infusion of 1% v/v EtOH (30 µl in 2 s). This triggered deployment of a second bottle containing water for 10 seconds, during which licks from the water spout were recorded.

### Training and Drug Schedule

After surgical recovery, rats were overnight water deprived for continuous access training. For 2 days, rats received 1 hr water access in the operant chambers and 3 hrs of water access each afternoon in their home cages to ensure proper hydration. Following 2 days of water training, rats began daily EtOH self-administration sessions of 1 hr duration. Rats were placed in the operant chambers with three spouts: spout 1 (left – “water” spout), spout 2 (middle – “active” spout) and spout 3 (right – “inactive” spout) were empty. Upon program activation, empty spouts 2 and 3 were presented, with licks on the inactive spout producing no programmed consequences and licks on the active spout counting towards completion of the FR5 schedule of reinforcement. Following 12 days of FR5 acquisition and maintenance, the schedule was changed to a PR2 requirement, where requirement for EtOH access increased by 2 licks per reinforcement (PR2: i.e. 2, 4, 6, etc.). If subjects did not meet the scheduled requirement after 10 minutes, the session was terminated without a reinforcement reward, providing the animals’ breakpoint (defined as the number of reinforcement cycles completed). Assuming the subject reached the active spout requirement, the water spout was presented for a 10 s interval, during which licks were recorded. At the end of the 10 s interval, the spout retracted and the procedure was repeated.

### Data Analysis

Body weight (g) and food intake (kcals) were measured daily, are presented as Mean ± SEM of Group (RYGB or SHAM), and analyzed using two-way factorial ANOVA with Group (RYGB or SHAM) and Day as independent factors.

During experiment one and two, FR5 responses and PR2 responses, respectively, for 1% IV EtOH were measured and presented as Mean ± SEM of Group (RYGB or SHAM, each n = 6). For each individual experiment, the number of infusions and licks made on all water spouts were measured and analyzed as dependent factors using two-way factorial ANOVAs with Group (RYGB or SHAM) and Day as independent factors. All significant findings were further analyzed using Fisher’s LSD post hoc testing. All analyses were conducted using Statistica 8.0 (StatSoft, Inc; Tulsa OK).

## Results

### Body Weight and Food Intake

Preoperative body mass for RYGB and SHAM was 636.84±61.30 g, and 611.42±11.13 g, respectively (NS). At the beginning of the EtOH self-administration, RYGB rats weighed 594.3±43.0 g, while SHAM rats weighed 734.7±32.7 g. During the experiment, body weight was significantly lower in RYGB rats. We found a significant effect of Group (F(1,112) = 18.610, p<0.001), but not Day (F(13,112) = 0.075, p = 0.99), nor a Group by Day interaction (F(13,112) = 0.050, p = 1.00) on body weight. Post hoc testing revealed RYGB rats had a lower body weight across all days of testing (p<0.01). We also measured daily high fat diet intake by RYGB and SHAM groups, in kilocalories consumed across a 24 hr period (kilocalories from alcohol consumption not included). ANOVA revealed no significant effect of Group (F(1,112) = 1.541, p = 0.22) or Day (F(13,112) = 1.653, p = 0.08), nor a Group by Day interaction (F(13,112) = 0.620, p = 0.83).

### Experiment One: FR5 Responses to 1% IV EtOH in RYGB and SHAM Rats

**Figure 1:**
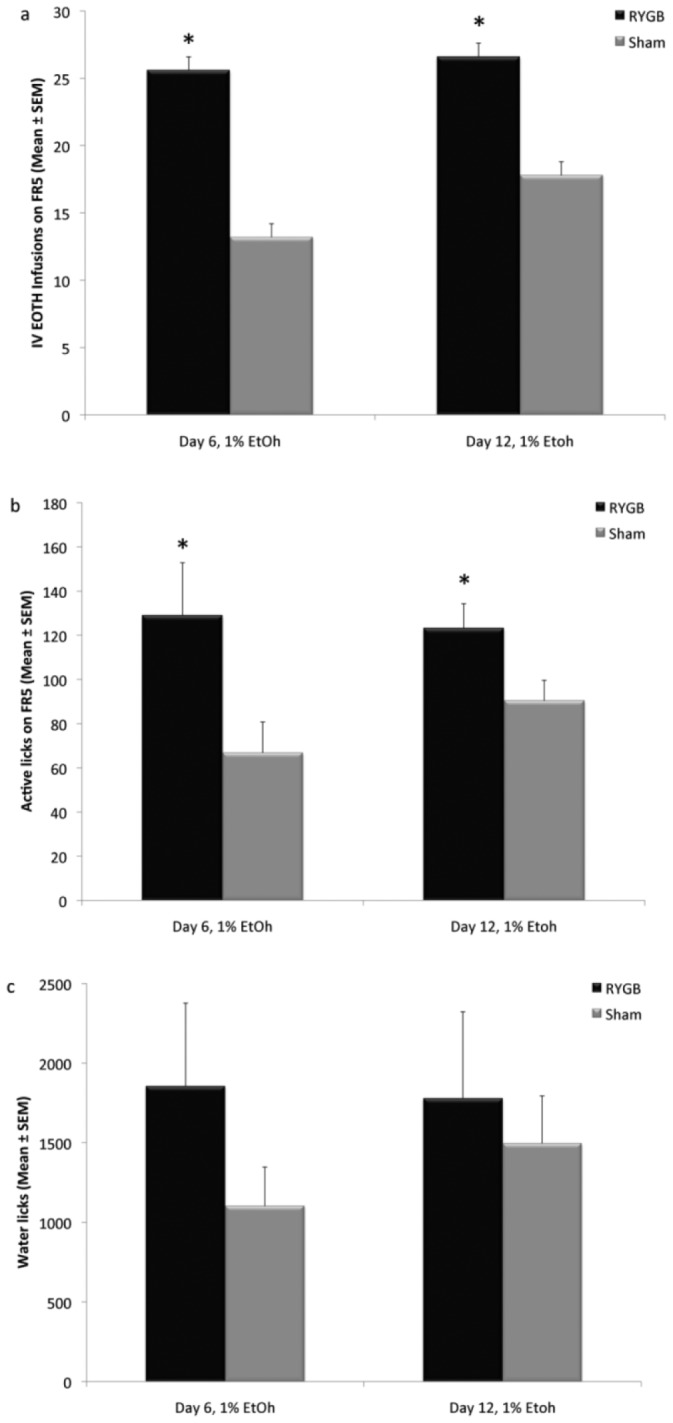
RYGB rats (n = 6) worked harder for, and infused more IV ethanol during FR self-administration sessions than SHAM controls (n = 6). A) IV EtOH infusions were averaged and presented as Mean ± SEM. RYGB rats infused significantly more IV EtOH on days 6 and 12. B) Active spout licks, presented as Mean ± SEM. RYGB rats made more licks on the active spout than Sham rats on days 6 and 12. C) Number of active licks on the water spout, presented as Mean ± SEM. There were no significant differences between groups at any time point. * p<0.05.

RYGB rats made significantly more EtOH infusions and more licks in the active spout than SHAM rats (Fig. 1). Two-way ANOVA showed an effect of Group (F(1,96) = 13.9582, p<0.001), but not Day (F(11,96) = 0.9605, p = 0.487), or a Group by Day interaction (F(11,96) = 0.3817, p = 0.960). Post hoc testing revealed RYGB rats obtained significantly more EtOH infusions compared to their SHAM counterparts on Day 6 (p<0.05) and Day 12 (p<0.05) (Fig. 1a). ANOVA also revealed a significant effect of Group (F(1,96) = 13.4288, p<0.001), but not Day (F(11,96) = 0.8571, p = 0.584), or a Group by Day interaction (F(11,96) = 0.3868, p = 0.958) on active licks. Post hoc testing revealed RYGB rats made significantly more licks on the active spout on Day 6 (p<0.05) and Day 12 (p<0.05) (Fig. 1b).

The number of licks on the water spout did not differ between Groups (F(1,96) = 1.2940, p = 0.2600), Day (F(11,96) = 1.4201, p = 0.1764), or a Group by Day interaction (F(11,96) = 0.5444, p = 0.8683) (Fig. 1c). Licks made on the inactive spout did not differ as an effect of Group (F(1,96) = 2.2767, p = 0.1346) or a Group by Day interaction (F(11,96) = 0.3945, p = 0.9552). There was a significant effect of Day (F(11,96) = 2.2359, p<0.05). Post hoc analysis revealed a significant effect in RYGB rats (all: p<0.001) and Sham rats (p<0.05) on the first day of testing (Day 1) compared to the following days. These effects are attributable to acclimation and initial learning taking place on the first day of testing during the FR5 schedule of reinforcement.

### Experiment Two: PR2 Responses to 1% IV EtOH in RYGB and SHAM Rats

**Figure 2:**
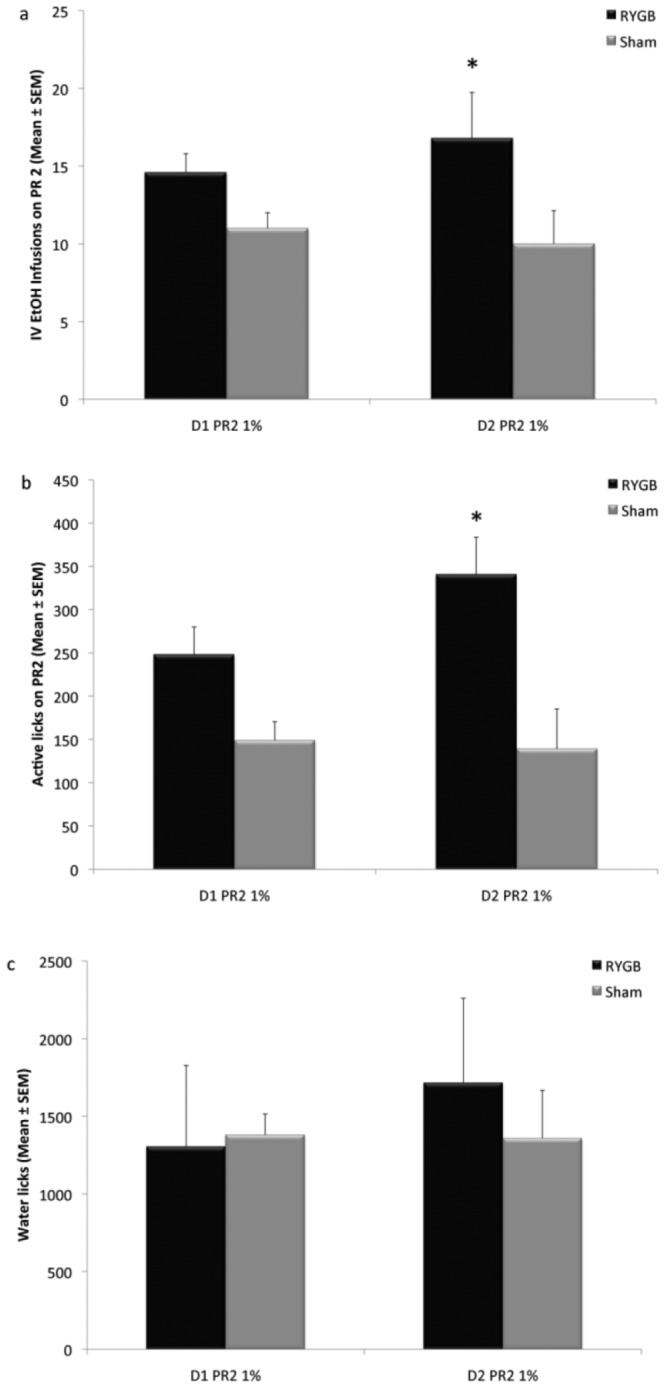
RYGB rats (n = 6) worked harder for, and infused more IV ethanol during PR self-administration sessions than SHAM controls (n = 6). A) IV EtOH infusions were averaged and presented as Mean ± SEM. RYGB rats infused significantly more IV EtOH on day 2 of PR testing (D2 PR2 1%), achieving higher breakpoints than Sham controls. Though RYGB rats also infused more IV EtOH on day 1 this did not reach significance (D1 PR2 1%). B) Active spout licks, presented as Mean ± SEM. RYGB rats made more licks on the active spout than Sham rats on PR day two. C) The number of active licks on the water spout, presented as Mean ± SEM. There were no significant differences between groups at any time point. * p<0.05.

The number of infusions earned by RYGB and SHAM rats differed (Figure 2a) showing a significant Group effect (F(1,16) = 6.9333, p<0.05) but not Day (F(1,16) = 0.0923, p = 0.7652), nor Group by Day interaction (F(1,16) = 0.6564, p = 0.4297). Post hoc analysis revealed that RYGB rats earned significantly more infusions on the second day of PR testing than SHAM rats (p<0.05).

The number of active licks also differed by Group (F(1,16) = 5.9821, p<0.05), but not Day (F(1,16) = 0.2293, p = 0.6386), or a Group by Day interaction (F(1,16) = 0.8709, p = 0.3646) (Figure 2b). Post hoc analysis revealed RYGB rats made significantly more active licks on the second day of PR testing than SHAM rats (p<0.05).

ANOVA revealed no significant effect of water licks by Group (F(1,16) = 0.1145, p = 0.7395), Day (F(1,16) = 0.2150, p = 0.6491), or a Group by Day interaction (F(1,16) = 0.2659, p = 0.6132) (Figure 2c). ANOVA also revealed that there was no significant effect on inactive licks by Group (F(1,16) = 0.0003, p = 0.9855), Day (F(1,16) = 0.3295, p = 0.5739) or a Group by Day interaction (F(1,16) = 0.6943, p = 0.4170).

## Discussion

Obese rats after RYGB self-administered more EtOH and had higher breakpoints than SHAM obese controls. Since the EtOH was administered IV, this provides evidence that the rewarding effects of EtOH were not due to increased absorption following RYGB.

RYGB rats displayed a significant increase in EtOH-seeking behaviors compared to SHAM controls. Not only did this effect reach significance on days 6 and 12 (midway through and at the end of our procedure), but also RYGB rats compared to controls expended more efforts to earn EtOH reward and displayed increased EtOH consumptions even on days when differences did not reach statistical significance. RYGB rats also worked harder than SHAM when switched to a PR schedule of reinforcement, resulting in significant difference on Day 2. Whereas the inherent difficulties with IV EtOH self-administration [20], [21] and individual differences in surgical outcomes might explain the varying magnitude of the effect, the fact that RYGB rats were willing to work harder for IV EtOH reward under both schedules of reinforcement provides consistent evidence of increased susceptibility to EtOH’s reinforcing effects. Thus, although the literature shows that RYGB reduces the rewarding effect of certain foods, the opposite may be true for other rewarding substances such as alcohol.

Our current findings not only are consistent with previous ones investigating the effects of RYGB on oral EtOH intake [15], [16], [17], but also represent the first demonstration of the effects of RYGB on IV EtOH administration. An important advantage of the IV self-administration procedure is that it permits investigation of the direct and central reinforcing effects of EtOH, without interference from peripheral orosensory and gastrointestinal (GI) factors, as could be expected with oral EtOH delivery. Thus, these results suggest that higher alcohol intake in RYGB rats is not due to oral and postoral factors (e.g. taste, GI-chemosensation and peptide release). Rather, it is likely that RYGB alters the sensitivity to alcohol reward, presumably through effects in the mesolimbic dopamine system. One possibility is that RYGB may improve the sensitivity of the dopamine system, which might be blunted in obesity [22]. Indeed RYGB in humans was shown to alter dopamine D2 receptors in the ventral striatum [23], [24], a brain region implicated in alcohol’s rewarding effects [25]. Additionally, our findings most likely reflect post-RYGB metabolic and endocrine changes that impact the sensitivity of the brain to alcohol reward. Notably, hormones that have been shown to change after RYGB, such as leptin and ghrelin [26], [27], [28], are also known to modulate the dopaminergic reward system [29], [30], [31] as well as EtOH consumption [32], [33]. Thus, it is conceivable that RYGB may reverse blunted ghrelin signaling in obesity [34], [35], which acting upstream on the dopamine neurons [36], [37], [38], may alleviate reward deficits associated with dietary obesity [39]. In fact, modest changes in ghrelin sensitivity may exert behaviorally relevant effects [40], and might be a contributing factor in increased alcohol reward following RYGB [16]. Similarly, transcriptional changes in orexin and dopamine systems implicated in the regulation of both food and ethanol reward have been shown in DIO rats following RYGB [17] further supporting the notion that increased postsurgical EtOH consumption, in part, is driven by an augmented reward response.

Our findings support recent clinical investigations showing increased susceptibility to alcohol abuse following RYGB [9], [10], [41], [42] and are further corroborated by our recent studies that show increased alcohol seeking, taking, and preference in DIO rats that received RYGB [15], [16]. It is worth noting that our results differ from those in a recent study that showed decreased EtOH intake in alcohol-preferring rats, and decreased alcohol intake after RYGB in obese patients that regularly drank alcohol prior to undergoing the surgery [43]. However, a more recent study by the same group corroborates our previously published work and current findings [17]. While the factors that contribute to the discrepancies between the earlier studies by Davis and colleagues are unclear, it may reflect, at least in part, the role of genetics and/or pre-surgical EtOH use history in the response to alcohol reward after RGYB. Specifically, Davis and colleagues tested rats that were bred for their preference to drink alcohol; similarly, their clinical results showed that patients who frequently used alcohol were those in whom RGYB induced the greatest decreases in alcohol intake. In contrast, King and colleagues [42] reported that 7–9% of patients who have never displayed ethanol abuse begin to drink following RYGB. These data are supported by the preclinical findings in rodents with no prior history of EtOH exposure [15], [16], [17]. Thus genetic predisposition cannot entirely account for differences in EtOH intake between these two populations following RYGB. It is possible that differing clinical populations and procedural differences may explain these differential results. Additional clinical and pre-clinical studies are warranted to determine susceptibility factors that may lead to increased alcohol consumption in some bariatric patients.

Though the two groups of rats did not differ in preoperative body weight, the RYGB rats weighed significantly less than SHAM counterparts after the surgical procedure. This is expected from the surgical procedure and consistent with the weight loss reported following RYGB in rats [6], [8], [15], [27]. Caloric restriction and deprivation are potent enhancers of food and drug reward [44], [45]. Therefore, one of the potential mechanisms responsible for the increased EtOH reward after RYGB is caloric restriction. In their recent study using high fat diet-induced obese rats, Davis *et al*. [17] tested this notion using a control group that was food-restricted for approximately 20 days to mimic postsurgical weight loss in the obese RYGB group. They found no effect of weight loss on EtOH intake in the weight-reduced group, while lean rats displayed increased EtOH intake following the RYGB procedure. This observation suggests that the effect of RYGB to increase EtOH intake occurs independent of changes due to body weight loss. Based on this finding, a restricted-fed control group was omitted from the present study. Despite the lower body weight, however, the RYGB rats consumed as much food as SHAM, probably reflecting surgery-induced malabsorption including impaired absorption of fat [46]. Furthermore, increased metabolic rate following RYGB in both human and animal models [2], [47], [48] may also explain the observed discrepancy between changes in food intake and body weight. In addition, clinical studies have found no correlation between increased alcohol use and the degree of weight loss [49]. Also, distinct to RYGB, patients that underwent a laparoscopic adjustable gastric band did not increase alcohol use [42], which indicates that the increased alcohol intake is not likely a compensatory response to impaired nutrient and calorie absorption. Nevertheless, factors driving EtOH consumption are likely to include both its rewarding effects as a nutrient (delivering calories) and its pharmacological effects (increasing dopamine and endogenous opiates). Whereas in the present study the infused amount of EtOH had negligible caloric content, follow-up studies using weight-matched or pair-fed control groups are warranted. Similarly, future studies are required to determine the role of dietary fat in increased EtOH intake following RYGB. In fact, high-fat fed rats have been reported to display abnormally low motivation to work for natural rewards in certain operant tasks (e.g. [50]). Thus, fat malabsorption after RYGB is a plausible factor that could alleviate reward deficits following chronic high fat food consumption.

It is also possible that altered EtOH metabolism and clearance following RYGB [11], [12], [13], [14] might influence EtOH reward. To determine if increased EtOH consumption following RYGB surgery was due to altered blood alcohol concentration (BAC), Davis *et al.* [17] measured BAC 30 min following oral gavage of EtOH approximating the dose spontaneously consumed by the rats. RYGB rats displayed similar BAC compared to sham surgery and non-surgery control rats, which indicates that differences in alcohol metabolism do not account for the changes in reward after RYGB. For this study, we used a low concentration of EtOH (1%), which we chose based on previous operant IV self-administration studies [21], [51]. We chose this low dose since IV administration of EtOH results in significantly greater BAC than the same dose administered orally [52], and the rapid intoxication and sedative effects of higher doses would interfere with the performance of the rat on the progressive ratio task. Furthermore, in the rats, dopamine in the nucleus accumbens shell increases in a dose-dependent manner between 0.5–1.0 g/kg doses, but the response to higher EtOH doses reaches a plateau [20]. Although BAC was not directly assessed in our study, the total cumulative IV dose self-administered over a 30-min time period should have resulted in lower BAC than the intragastric bolus (0.5 g/kg EtOH) used in the Davis *et al.* study [17]. Thus, as with oral self-administration, it is unlikely that difference in BAC, if any, between RYGB and SHAM were contributory to increased EtOH reward in our study. In fact, RYGB rats self-administered significantly more infusions than SHAM in both the FR and the PR tests regardless of the total amount of EtOH received (average infusions of about 25 on FR vs. 15 on PR). Another possible confound was that the rats in our study were water deprived, and working for EtOH reinforcement was accompanied by deployment of the water spout in our operant chambers. However, despite a trend for increased water intake by RYGB corresponding with previous observations [15], there were no significant differences between RYGB and SHAM rats in water intake throughout the sessions. It is possible that we were underpowered to detect small differences between the groups. Moreover the significant effect of RYGB on alcohol but not on water intake indicates that overall increases in fluid consumption does not account for our results (Figs. 1c & 2c).

In summary, we show that RYGB rats display greater EtOH IV self-administration compared to dietary obese controls. These findings support previous clinical reports of increased susceptibility to alcohol abuse in bariatric patients. Reward-related and neuroendocrine mechanisms are likely involved in RYGB-related increase in alcohol reward, as we bypassed the normal route of alcohol ingestion and directly assessed reward via IV self-administration. Further research is required to confirm translation of these preliminary findings to humans and to determine underlying mechanisms, which, in turn, might result in personalized interventions and treatments.
